# Distraction and Mind-Wandering Under Load

**DOI:** 10.3389/fpsyg.2013.00283

**Published:** 2013-05-22

**Authors:** Sophie Forster

**Affiliations:** ^1^Department of Clinical, Educational and Health Psychology, University College LondonLondon, UK

**Keywords:** attention, distractor interference, irrelevant distraction, mind-wandering, perceptual load, task-unrelated thought

## Abstract

Attention research over the last several decades has provided rich insights into the determinants of distraction, including distractor characteristics, task features, and individual differences. Load Theory represented a particularly important breakthrough, highlighting the critical role of the level and nature of task-load in determining both the efficiency of distractor rejection and the stage of processing at which this occurs. However, until recently studies of distraction were restricted to those measuring rather specific forms of distraction by external stimuli which I argue that, although intended to be irrelevant, were in fact task-relevant. In daily life, attention may be distracted by a wide range of stimuli, which may often be entirely unrelated to any task being performed, and may include not only external stimuli but also internally generated stimuli such as task-unrelated thoughts. This review outlines recent research examining these more general, entirely task-irrelevant, forms of distraction within the framework of Load Theory. I discuss the relation between different forms of distraction, and the universality of load effects across different distractor types and individuals.

The experience of being unintentionally distracted from an intended focus is likely to be frustratingly familiar to most people, and such distraction can prove highly disruptive in a variety of daily life contexts (e.g., education, Rabiner et al., 2004; in the workplace, Wallace and Vodanovich, 2003; or while driving, Arthur and Doverspike, 1992). Over the past decades a large body of research has investigated the determinants of the ability to focus attention on relevant stimuli, while avoiding distraction from irrelevant stimuli, highlighting a number of important factors. These include features of the distractor such as visual salience or abrupt onset in the display (e.g., Theeuwes, 1992; Yantis, 2000) and individual differences (e.g., in working memory capacity (WMC); Kane and Engle, 2003). The level of perceptual load in a task has been identified as a particularly powerful determinant of distraction: according to the Load Theory (e.g., Lavie, 1995, 2005, 2010), irrelevant (and potentially distracting) stimuli can only be perceived if there is sufficient spare perceptual capacity left over from task processing. Distraction can therefore be reduced or altogether avoided during more perceptually demanding tasks, which fully exhaust perceptual capacity and so reduce or prevent distractor processing. In contrast, tasks which impose only a low level of perceptual load leave spare capacity, which allows processing of potentially distracting non-task stimuli.

In support of Load Theory, increased perceptual load (in terms of a greater number of task stimuli requiring processing, or more complex perceptual task demands) has been found to reduce both the visual-cortical response to irrelevant stimuli (e.g., Yi et al., 2004; Schwartz et al., 2005), and a range of behavioral indices of distractor processing including response-competition (e.g., Lavie, 1995; Lavie and Cox, 1997), negative priming (Lavie and Fox, 2000), and inattentional blindness (Cartwright-Finch and Lavie, 2007). However, as I shall discuss, until recently empirical studies of perceptual load effects, and of distraction in general, were limited to those using external distractor stimuli that were in some way relevant to the task being performed. Load Theory implies that under low load even entirely task-irrelevant stimuli will be processed and could potentially (providing that they are of sufficient salience) cause distraction. Indeed, in daily life, people may often be distracted by stimuli seemingly entirely unrelated to the task that they are currently engaged in – for example a student may be distracted from studying by the sight of a friend walking by. In addition, task-irrelevant distractions may come not only from the external environment but also from internally generated stimuli associated with mind-wandering – for example, a student may be distracted from reading an assigned article by the intrusion of a thought about an unrelated issue – perhaps some salient recent event in his or her daily life. In the following sections I consider the extent to which both well established and more recent laboratory measures address the common daily life experience of entirely task-irrelevant distraction (by both internal and external stimuli), and discuss recent studies extending Load Theory to these forms of distraction.

## Established Measures of Distraction

**Figure 1:**
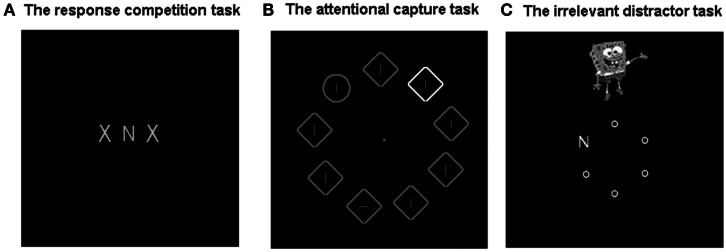
**Measures of distractor interference: example displays**. **(A)** The response-competition-task. In this task participants make forced-choice responses to a target item (in this example, either X or N). Distraction is indexed by the RT increase when the target item is flanked by distractors representing the competing response (pictured) versus those representing the same response. **(B)** The attentional capture task. In the typical attentional capture task, distraction is indexed by the increase in search RTs for a target item (in this example a circle), when one of the non-target search items appears as a salient singleton in an irrelevant dimension (e.g., color), compared to a no singleton baseline. **(C)** A new measure of interference from salient yet entirely task-irrelevant distractors. Within these measures distraction is indexed by the increase in RTs associated with the peripheral presentation of a colorful distractor. This can be either an image of a well known cartoon character (selected from Superman, Spiderman, Pikachu, Spongebob Squarepants, Micky Mouse, and Donald Duck) or meaningless yet colorful shape.

A widely used measure of distraction is the response-competition task (e.g., Eriksen and Eriksen, 1974; see Figure 1A for example). Within this task, participants are slowed in responding to targets in the presence of response-incompatible versus response-compatible distractors. In contrast to predecessors such as the Stroop task (Stroop, 1935), the target and distractors are presented in spatially separate locations which are known to the participant. As the target location is known, participants have no reason to search the distractor locations, making these locations entirely irrelevant.

In this way the response-competition task appears to reflect situations in daily life in which an individual is distracted by a stimulus appearing in an unattended location. However, although the location is irrelevant, the identity of response-competition distractors is highly relevant to the task. In the most typical versions of the task the distractor stimuli are of the same type as target (e.g., both are letters), although some versions of the task use different stimulus types (e.g., pictures versus names) as target and distractor (e.g., Young et al., 1986). Nevertheless, by the very nature of the response-competition task all variants of this task have in common a strong response-relevance born by the distractor to the target. Interestingly, it has been demonstrated that the expected locations of response-competition distractors in fact appear to receive advance attentional allocation (resulting in speeded perception of other stimuli appearing in these locations, Tsal and Makovski, 2006). In these respects, the response-competition task differs somewhat from the kind of interference often experienced in daily life, from a distractor (e.g., a friend walking past) that is entirely unrelated to the task being performed (e.g., studying).

The question as to whether any task-irrelevant stimuli can nevertheless attract and distract attention has in fact been the focus of a contentious debate for some time, triggering the development of another widely used class of distraction measure: the Attentional Capture Paradigm (see Figure 1B for example). Using variants of this task, reaction time (RT) interference has been demonstrated in the presence (versus absence) of certain types of distractor, such as salient feature singletons (e.g., Theeuwes, 1991a, 1992) and abrupt onsets (e.g., Remington et al., 1992), even when these are response-irrelevant and visually distinct from the target stimuli. However, proponents of “contingent capture” have challenged studies purporting to show attentional capture from irrelevant stimuli, highlighting that even apparently task-irrelevant distractors may in fact be relevant to attentional settings for the task (e.g., Folk et al., 1992, 2002), and moreover, their ability to interfere may depend on this task-relevance. For example, interference from singleton distractors may be contingent on their relevance to a “singleton detection” search strategy adopted when the search target is also a singleton (even in a different dimension – e.g., color versus form; Bacon and Egeth, 1994). Task-relevance may also be conferred by more general aspects of the stimulus display: Gibson and Kelsey (1998) have argued, for example, that any task involving an onset of the stimulus display at the start of each trial may create “display-wide” attentional settings for abrupt onset stimuli, including distractors.

In addition, studies designed to demonstrate distraction by stimuli irrelevant to any attentional settings have primarily used search tasks in which the distractors appear in task-relevant locations, in or around potential target locations. As the specific target location is typically unknown, participants would be likely to allocate their attention diffusely across the entire display, including the locations in which the distractors were to appear. In the light of previous evidence suggesting that distractor effects can be eliminated with prior knowledge of location (Yantis and Jonides, 1990; Theeuwes, 1991b), it seems likely that location-relevance contributes to the distractor interference measured by such paradigms. A smaller number of studies (Christ and Abrams, 2006; Neo and Chua, 2006) have demonstrated attentional capture by abrupt onsets within paradigms in which the target location is known. However, even in these cases the location was not in fact entirely irrelevant – distractors and other non-targets were perceptually grouped with the target around fixation, which would have made them harder to ignore (see Driver and Baylis, 1989; Kramer and Jacobson, 1991).

## Irrelevant Distraction: External Sources

**Figure 2:**
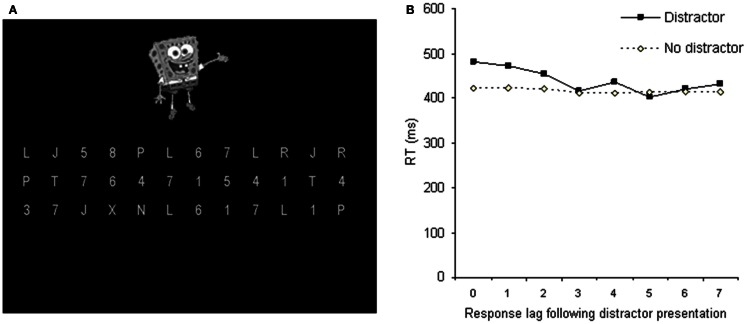
**A continuous task designed to preclude general attentional settings associated with the onset of the display**. **(A)** Example stimulus display: participants make sequential responses, working from left to right, top to bottom, indicating whether each item in the display is a letter or a digit. The display remains onscreen throughout the response sequence. The distractor appears briefly during a minority of displays, and never co-occurs with the responses immediately following the display onset. **(B)** Despite being entirely irrelevant to the task in terms of visual appearance, meaning, location, and any attentional settings, the brief presentation of a distractor produces significant RT slowing for the response immediately following its presentation (lag 0), and for the two subsequent responses (lags 1 and 2).

The studies reviewed above highlight that in order to be considered entirely task-irrelevant, distractors must be unrelated to any task responses, presented in an irrelevant location, visually dissimilar from the search stimuli and irrelevant to any attentional settings for the current task. A recent series of studies by Forster and Lavie (2008a,b, 2011) (see Figure 1C) introduced a new measure designed to meet these criteria. These studies have demonstrated robust RT slowing in the presence, versus absence, of a colorful distractor image (e.g., of the cartoon character Spiderman) across two different task types: a letter search (Forster and Lavie, 2008a,b) and a sequential forced-choice response task (Forster and Lavie, 2011; Figure 2). Irrelevant distractor interference has been found for meaningless (a colorful shape) and frequently presented (50% trials) stimuli, but was greater for semantically meaningful (e.g., a famous cartoon character) and infrequently presented (10% trials) stimuli (Forster and Lavie, 2008b, see also Biggs et al., 2012 for further examination of effects of meaningfulness on irrelevant distraction).

Note that in these studies, the complex and colorful distractor stimuli bore no visual similarity to the task stimuli (gray letters or digits), appeared in an irrelevant peripheral location, and were unrelated in content to any aspect of the task being performed. Although the distractor was a type of singleton (being the only stimulus of its kind in the display), the interference does not appear to depend on a use of a singleton detection search strategy as it persists even when such a strategy is unavailable (using a search set size of three; Forster and Lavie, 2008a,b). In addition, the brief onset of the irrelevant distractor during a novel sequential response task (see Figure 2) produced RTs slowing of up to three responses following its presentation. As the display in this task remained constant over multiple (9 or 36) responses, such interference cannot be attributed to attentional settings associated with onset of, or other dynamic changes to, the task stimuli. Thus, as in daily life, the distractors in these studies appear to interfere despite being entirely task-irrelevant.

Forster and Lavie (2008a) recently clarified that although interference from these salient and meaningful abrupt onset distractors persists in the absence of any task-relevance, it can be modulated by perceptual task-load. This study employed a widely used manipulation of load with a letter search task, whereby a letter search target is presented among non-targets that are either visually dissimilar (e.g., small circles, low load, see Figure 1C) or similar (e.g., other angular letters, high load) to the target. I note that this manipulation of load within response-competition tasks has recently been argued to reduce interference not via load, but via low level “dilution” effects whereby feature representations of the visually similar non-targets degrade the distractor representation (e.g., Tsal and Benoni, 2010; Wilson et al., 2011). Unlike response-competition letter distractors, however, the irrelevant distractors have very minimal feature overlap with the non-target stimuli in either the high or low load conditions. It appears less plausible that the inclusion of small, monochromatic letters (versus small, monochromatic circles) in the display would substantially degrade the representation of a larger, colorful cartoon image. Thus, the finding that the robust irrelevant distractor interference seen under low load can be reduced to non-significant levels under high load provides compelling evidence in support of the perceptual load hypothesis.

## Irrelevant Distraction: Internal Sources

In daily life sources of distraction may not only be found in the external environment, but also in the form of internally generated distractions such as task-unrelated thoughts (TUTs). Studies of mind-wandering suggest that this may be a highly disruptive form of distraction: increased reports of TUTs have been associated with impaired performance on a wide range of tasks from simple signal detection to more complex tasks such reading comprehension, listening to lectures, SAT examinations, and driving (Schooler et al., 2004; Smallwood et al., 2007; He et al., 2011; Risko et al., 2012; Unsworth et al., 2012).

Despite its apparent ubiquity in daily life, irrelevant distraction from task-unrelated mind-wandering has been largely neglected by studies of selective attention – perhaps due to the inherent difficulty in directly measuring such a subjective phenomenon. However, the growing literature on mind-wandering has established a number of measures, such as diary-keeping, questionnaires, or intermittent “thought-probing” during a task (see Smallwood and Schooler, 2006, for review), and recent individual differences research using these measures suggests that distraction from mind-wandering and external stimuli may be driven, at least in part, by common mechanisms. Kane and colleagues have argued that the ability to exert attentional control over mind-wandering draws on an executive control mechanism (e.g., McVay and Kane, 2010), which also supports attentional control over external stimuli (e.g., during Stroop or response-competition tasks, Kane and Engle, 2003; Levinson et al., 2012; Shipstead et al., 2012). In support of this claim, lower executive WMC has been linked to increased mind-wandering (e.g., Kane et al., 2007; McVay and Kane, 2009). Consistent with the notion of a role of WMC in avoiding distraction from mind-wandering, this relationship has been found to be strongest during tasks that participants classified as requiring concentration (Kane et al., 2007).

A more direct link between internal and external forms of distraction was made in a recent study (Forster and Lavie, 2013) examining the relation between individual differences in mind-wandering and two measures of external distraction: response-competition interference, and our recently established measure of entirely irrelevant distraction (as described above; Forster and Lavie, 2008a,b). In two experiments, individuals who reported higher levels of daily life mind-wandering also showed increased RT interference from task-irrelevant external distractors. However, this study highlighted that not all forms of distraction are alike: mind-wandering was not related to response-competition interference in either experiment. Moreover, interference from response-competition letter distractors was unrelated to our measure of task-irrelevant distractor interference. Thus, this study suggests a common trait specifically underlying the ability to ignore entirely irrelevant stimuli, regardless of whether these are internal (i.e., TUTs) or external, while also highlighting the importance of task-relevance in determining distraction.

An interesting question is whether, in addition to (in some cases) drawing on a common trait, internal, and external forms of distraction also share the common determinant of perceptual load. Recent studies (Forster and Lavie, 2009; Levinson et al., 2012) have examined this issue: during a letter search task with high and low perceptual load, participants were intermittently probed as to whether their current thought was task-related or task-unrelated. In keeping with the well established effects on external distraction, reports of TUTs were reduced with the increase in perceptual load. Moreover, one experiment incorporating both thought probes and response-competition distractors (Forster and Lavie, 2009, Experiment 4) demonstrated that the extent of load effects on these two forms of distraction was correlated between individuals. Thus, both internal and external forms of distraction appear subject to modulation by a common mechanism, depending on the level of perceptual load in the current task.

I note that the substantial qualitative differences between response-competition distractors and TUTs make it somewhat implausible that this common mechanism involves low level “dilution” of both types of distractor representation by the letter non-targets: indeed, it is difficult to conceive of a situation in which the representation of a TUT (e.g., involving salient current concern, Smallwood and Schooler, 2006) would be diluted simply by the presence of five externally presented monochromatic letters. Rather, the results of this study appear in line with the suggestion that when perceptual capacity is exhausted by task demands, vulnerability to interference from potential distractors is reduced regardless of whether these are internal or external.

## How Universal are Perceptual Load Effects on Distraction?

Perceptual load is well established to modulate interference from response-competition distractors, whether these are presented in irrelevant peripheral locations (e.g., Lavie, 1995; Lavie and Cox, 1997), or fixation (Beck and Lavie, 2005); and whether these are simple letters as per the traditional response-competition task, or meaningful images (Lavie et al., 2003). The studies described above extend Load Theory to forms of distraction (both internal and external) which produce robust interference despite their irrelevance to the current task. The common effect of perceptual load on mind-wandering and response-competition interference is particularly striking given that these two forms of distraction do not appear to be directly correlated with each other (Forster and Lavie, 2009, 2013). This suggests that load effects may be universal across distractor types, regardless of their task-relevance or their relation to each other. Indeed, neuro-imaging findings suggest that perceptual load can also reduce processing even of potentially biologically important yet irrelevant stimuli, such as the amygdala response to threat (Bishop et al., 2007) and motion processing in V5 (Rees et al., 1997), as well as behavioral interference from moving or abrupt onset distractors (Cosman and Vecera, 2009, 2010).

Interestingly, the one potential exception to perceptual load effects appears to be distractor stimuli with which participants have a high degree of familiarity or expertise: response-competition interference from famous faces and musical instruments among musicians (but not non-musicians), as well as interference from task-irrelevant national flags or sports team logos, has been found to persist under high perceptual load (Lavie et al., 2003; Ro et al., 2009; Biggs et al., 2012). Thus, when stimuli have a high degree of personal relevance, they may be prioritized for processing regardless of perceptual load or task-relevance.

Perceptual load effects also appear to be largely universal across individuals, with one important exception: as load effects depend on capacity limits, individual differences in perceptual capacity (e.g., those associated with age, Maylor and Lavie, 1998; Huang-Pollock et al., 2002; video game expertise, Green and Bavelier, 2003; or conditions such as autism or congenital deafness, Proksch and Bavelier, 2002; Remington et al., 2009) lead to differences in the level of load required to reduce distraction. However, factors predicting vulnerability to distraction, such as self-reported daily life attentional failures, trait anxiety, and WMC, have been found to do so only during tasks with low load, and not high load (Bishop et al., 2007; Forster and Lavie, 2007; Bishop, 2009; Levinson et al., 2012).

## Conclusions

The findings discussed here highlight the importance of considering the role of task-relevance in distraction. Although certain forms of distraction may be contingent on their task-relevance, studies using new measures demonstrate that task-relevance is not a necessary condition for distraction. Rather, as in daily life, sources of distraction may be entirely task-irrelevant, and may also include both external stimuli and task-unrelated mind-wandering. It is unclear to what extent these common, yet understudied, forms of distraction are directly related to other laboratory measures such as the response-competition task. However, perceptual load appears a powerful and largely universal determinant of distraction, across both existing measures and new measures of irrelevant distraction (both internal and external), as well as across individuals. Thus, Load Theory provides a useful framework for predicting when a variety of forms of daily life distraction are most likely to occur (i.e., during tasks with low perceptual complexity and demands) and even for interventions to prevent this (e.g., by increasing perceptual complexity).

## Conflict of Interest Statement

The authors declare that the research was conducted in the absence of any commercial or financial relationships that could be construed as a potential conflict of interest.
